# Is Consumption Disappearing

**Published:** 1897-04

**Authors:** 


### Is Consumption Disappearing.

Dr. Arthur Ransome, of Manchester, England, thinks that "if phythisis were to continue to diminish in prevalence at the same increasing rate of decline for another thirty years it would then have entirely disappeared. The first great drop in its rate took place in the decade] 1840 to 1850, about the time that serious attention began to be given to sanitary reform and especially to land drainage. It then remained scarcely reduced for about seventeen years; but from 1867 to 1894 it has been steadily on the decline. It is in this period that most of the great sanitary work has been carried on in this country." Surely here is an indication in what direction an effort should be made toward stamping out a desease which is said to carry off one-seventh of the human race. One would think insurance companies would be the first to promote a reform of this character, but they will not, their method of covering a risk is to charge an extra rate. There is no extra rate against death from consumption.—*Medical Examiner*.

